# Supplementary material for: The dynamic three-dimensional organization of the diploid yeast genome
Source: eLife. 2017 May 24;6:e23623. doi: 10.7554/eLife.23623 (PMC5476426; doi:10.7554/eLife.23623)
Supplement: Supplementary file 1. — DOI: http://dx.doi.org/10.7554/eLife.23623.025 [file elife-23623-supp1.docx]

**Supplementary file 1. Strains used in this study.**

| **Strain** | **Description** | **Source** |
| --- | --- | --- |
| FY5 (DBY11070) | *S. cerevisiae* *MAT*α | F. Winston |
| FY69 (YMD857) | *S. cerevisiae* *MAT***a** *leu2Δ1* | F. Winston |
| FY2 (DBY7283) | *S. cerevisiae* *MAT*α *ura3-52* *GAL*+ | F. Winston |
| BY4741 (YMD2177) | *S. cerevisiae* *MATa his3Δ1 leu2Δ0 met15Δ0 ura3Δ0* | SGRP |
| BY4742 (YMD2220) | *S. cerevisiae* *MAT*α *his3Δ1 leu2Δ0 lys2Δ0 ura3Δ0* | S. Fields |
| YMD1797 | *S. cerevisiae* *MAT*α *leu2Δ1* | C. Payen |
| YZB5-113 (YMD377) | *S. uvarum* *MAT*α *ho::Hyg^R^* *lys2-1* *ura3::Nat^R^* | Y. Zheng |
| YDG613 | *S. paradoxus* *MAT***a**/α | D. Greig |
| ILY376 (YMD3258) | *S. uvarum* *MAT***a** *ho::Kan^R^* *ura3::Nat^R^ LYS* | This study |
| Y12 (YMD1182) | *S. cerevisiae* *MAT***a** | J. Andrie (SGRP) |
| DBVPG6044 (YMD2218) | *S. cerevisiae* *MAT*α | J. Andrie (SGRP) |
| Nup60-TAP | *S. cerevisiae MATa* BY4741 *NUP60-TAP <HIS>* | (Ghaemmaghami et al., 2003) |
| ILY456 (YMD3259) | *S. cerevisiae* (*MAT*α) x *S. uvarum* (*MAT***a**) *Su ho::Kan^R^ Su ura3* | This study |
| YMD3263 | *S. uvarum* (*MAT***a**) ILY376 x *S. cerevisiae* (*MAT*α) BY4742 t(ScV;ScXII)(*YER151C*-*YER152C*;*YLR150W*-*YLR151C*) *ScYLR150W*-*ScYLR151C:Hyg^R^*-*URA3L*-*AI*-*lox*-*AI*-*URA3R*-*Nat^R^* *ScYER151C*-*ScYER152C:lox** | This study |
| YMD3264 | *S. cerevisiae* (*MAT*α) YMD1797 x *S. paradoxus* (*MAT***a**) sporulated YDG613 | This study |
| YMD3265 | *S. paradoxus* (*MAT***a**) sporulated YDG613 x *S. uvarum* (*MAT*α) YZB5-113 | This study |
| YMD3266 | *S. cerevisiae* (*MAT*α) x *S. uvarum* (*MAT***a**) ILY456 *Sc ngl2-ymr295cΔ::Hyg^R^* | This study |
| YMD3267 | *S. cerevisiae* (*MAT*α) x *S. uvarum* (*MAT***a**) ILY456 *Sc has1Δ::Hyg^R^* | This study |
| YMD3268 | *S. cerevisiae* (*MAT*α) x *S. uvarum* (*MAT***a**) ILY456 *Sc has1Δcoding::Hyg^R^* | This study |
| YMD3269 | *S. cerevisiae* (*MAT*α) x *S. uvarum* (*MAT***a**) ILY456 *Sc has1Δpr::Hyg^R^* | This study |
| YMD3270 | *S. cerevisiae* (*MAT*α) x *S. uvarum* (*MAT***a**) ILY456 *Sc has1prΔ::Hyg^R^* *YNL266W*-*YNL267W*::*HAS1pr*-*TDA1pr*-*Nat^R^* | This study |
| YMD3271 | *S. cerevisiae* Y12 (*MAT***a**) x *S. cerevisiae* DBVPG6044 (*MAT*α) | J. Andrie |
| YMD3377 | *S. cerevisiae* YMD1797 (*MATα*) x *S. uvarum* ILY376 (*MATa*) *Sc nup2Δ::HygR Sb nup2Δ::HygR* | This study |
| DBY827 | *S. cerevisiae MAT***a** *ade2-1 can1-100 his3-11,15 leu2-3,112 trp1-1 ura3-1 ADE2 HIS3:pAFS144 TRP1:pER04 HAS1:p6LacO128-HAS1* | This study |
| DBY828 | *S. cerevisiae MAT*α *ade2-1 can1-100 his3-11,15 leu2-3,112 trp1-1 ura3-1 ADE2 LEU2:p5LacI-GFP TRP1:pER04 HAS1:p6LacO128-HAS1* | This study |
| DBY830 | *S. cerevisiae MAT***a** *ade2-1 can1-100 his3-11,15 leu2-3,112 trp1-1 ura3-1 ADE2 mlp2Δ::Kan^R^ HIS3:pAFS144 TRP1:pER04 HAS1:p6LacO128-HAS1* | This study |
| DBY831 | *S. cerevisiae MAT***a***/*α *ADE2/ade2-1 can1-100/can1-100 HIS3:LacI-GFP/his3-11,15 LEU2:p5LacI-GFP/ leu2-3,112 TRP1:pER04 TRP1:pER04 ura3-1/ura3-1 HAS1:p6LacO128-HAS1/HAS1:p6LacO128-HAS1* | This study |
| DBY832 | *S. cerevisiae MAT***a** *ade2-1 can1-100 his3-11,15 leu2-3,112 trp1-1 ura3-1 nup100Δ::Kan^R^ HIS3:pAFS144 TRP1:pER04 HAS1:p6LacO128-HAS1* | This study |
| DBY834 | *S. cerevisiae MAT***a** *ade2-1 can1-100 his3-11,15 leu2-3,112 trp1-1 ura3-1 nup2Δ:: Kan^R^ HIS3:pAFS144 TRP1:pER04 HAS1:p6LacO128-HAS1* | This study |

SGRP, *Saccharomyces* Genome Resequencing Project (Liti et al., 2009)
