# Supplementary material for: The dynamic three-dimensional organization of the diploid yeast genome
Source: eLife. 2017 May 24;6:e23623. doi: 10.7554/eLife.23623 (PMC5476426; doi:10.7554/eLife.23623)
Supplement: Supplementary file 3. — DOI: http://dx.doi.org/10.7554/eLife.23623.027 [file elife-23623-supp3.docx]

**Supplementary file 3. Hi-C libraries.**

| **Strain(s)** | **Condition** | **Restriction Enzyme** | **Read Length** | **Read pairs** |
| --- | --- | --- | --- | --- |
| YMD1797 | raffinose | Sau3AI | 80 bp | 50,609,269 |
| YDG613 | exponential | Sau3AI | 80 bp | 17,996,489 |
| YZB5-113 | exponential | Sau3AI | 80 bp | 20,160,361 |
| Y12 | exponential | Sau3AI | 150 bp | 70,141,448 |
| DBVPG6044 | exponential | Sau3AI | 150 bp | 62,960,123 |
| FY69 + YZB5-113 | saturated | Sau3AI | 80 bp | 17,238,325 |
| FY69 + YDG613 | exponential | Sau3AI | 80 bp | 16,280,645 |
| Y12 + DBVPG6044 | exponential | Sau3AI | 150 bp | 63,418,065 |
| ILY456 | saturated | Sau3AI | 80 bp | 25,139,616 |
| ILY456 | saturated | HindIII | 80 bp | 8,268,022 |
| ILY456 | exponential | Sau3AI | 80 bp | 12,131,350 |
| ILY456 | exponential, rep 2 | Sau3AI | 80 bp | 20,007,389 |
| ILY456 | galactose | Sau3AI | 80 bp | 18,384,435 |
| ILY456 | nocodazole | Sau3AI | 80 bp | 24,915,498 |
| YMD3263 | saturated | Sau3AI | 80 bp | 25,866,783 |
| YMD3263 | exponential | Sau3AI | 80 bp | 26,592,214 |
| YMD3264 | saturated | Sau3AI | 80 bp | 62,902,987 |
| YMD3264 | exponential | Sau3AI | 80 bp | 24,502,214 |
| YMD3265 | saturated | Sau3AI | 80 bp | 41,615,076 |
| YMD3265 | exponential | Sau3AI | 80 bp | 28,280,637 |
| YMD3266 | saturated | Sau3AI | 80 bp | 23,981,947 |
| YMD3267 | saturated | Sau3AI | 80 bp | 23,045,668 |
| YMD3268 | saturated | Sau3AI | 80 bp | 13,268,816 |
| YMD3269 | saturated | Sau3AI | 80 bp | 11,219,451 |
| YMD3270 | saturated | Sau3AI | 80 bp | 18,929,190 |
| YMD3270 | exponential | Sau3AI | 80 bp | 14,620,886 |
| YMD3271 | saturated | Sau3AI | 150 bp | 73,586,493 |
| YMD3271 | exponential | Sau3AI | 150 bp | 64,048,246 |
| YMD3377 | saturated | Sau3AI | 80 bp | 26,199,952 |
| YMD3377 | exponential | Sau3AI | 80 bp | 27,502,371 |
| YMD3377 | galactose | Sau3AI | 80 bp | 14,678,416 |
| TOTAL |  |  |  | 948,492,382 |
